# *Schizophyllum commune* Reduces Expression of the SARS-CoV-2 Receptors ACE2 and TMPRSS2

**DOI:** 10.3390/ijms232314766

**Published:** 2022-11-25

**Authors:** Te-Kai Sun, Wen-Chin Huang, Yu-Wen Sun, Jeng-Shyan Deng, Liang-Hsuan Chien, Ya-Ni Chou, Wen-Ping Jiang, Jaung-Geng Lin, Guan-Jhong Huang

**Affiliations:** 1 School of Pharmacy, College of Pharmacy, China Medical University, Taichung 404, Taiwan; 2 Graduate Institute of Biomedical Sciences, School of Medicine, China Medical University, Taichung 404, Taiwan; 3 Department of Chinese Pharmaceutical Sciences and Chinese Medicine Resources, College of Chinese Medicine, China Medical University, Taichung 404, Taiwan; 4 Department of Food Nutrition and Healthy Biotechnology, Asia University, Taichung 413, Taiwan; 5 Department of Pharmacy, Chia Nan University of Pharmacy and Science, Tainan 717, Taiwan; 6 School of Chinese Medicine, College of Chinese Medicine, China Medical University, Taichung 404, Taiwan

**Keywords:** *Schizophyllum commune*, adenosine, SARS-CoV-2, ACE2, TMPRSS2

## Abstract

The current global pandemic of severe acute respiratory syndrome coronavirus 2 (SARS-CoV-2) of COVID-19 has infected hundreds of millions of people, killed millions, and continues to pose a threat. It has become one of the largest epidemics in human history, causing enormous damage to people’s lives and economies in the whole world. However, there are still many uncertainties and continued attention to the impact of SARS-CoV-2 on human health. The entry of SARS-CoV-2 into host cells is facilitated by the binding of the spike protein on the virus surface to the cell surface receptor angiotensin-converting enzyme 2 (ACE2). Furthermore, transmembrane protease serine 2 (TMPRSS2) is a host surface protease that cleaves and proteolytically activates its S protein, which is necessary for viral infection. Thus, SARS-CoV-2 uses the ACE2 receptor for cell entry and initiates the S protein using the protease TMPRSS2. *Schizophyllum commune* (SC) is one of the most widely distributed fungi, often found on the rotten wood of trees that has been found to have various health benefits, including anticancer, antimicrobial activity, antiparasitic, and immunomodulatory function. In this article, SC significantly diminished the expression ACE2 and TMPRSS2 protein in vitro and in vivo without cell damage. In addition, adenosine from SC was also proven in this experiment to reduce the ACE2 and TMPRSS2 expression. Thus, our findings suggest that SC and adenosine exhibit potential for the repression of SARS-CoV-2 infection via the ACE2 and TMPRSS2 axis.

## 1. Introduction

Severe acute respiratory syndrome coronavirus 2 (SARS-CoV-2) infection is the most severe epidemic that humans have experienced in decades, with high morbidity and mortality. Unusual measures have been forced on countries to try to reduce the impact of the disease, but they have had limited success, with spread mainly occurring through asymptomatic patients, and they often do not provide timely and accurate results [1]. Despite the recent development of vaccines and drugs, there are still many severe cases of coronavirus disease (COVID-19). Unfortunately, there is currently no standardized treatment, and the management of severe cases is very challenging; however, most require data from clinical trials before they can be used widely and safely [1,2].

Angiotensin-converting enzyme 2 (ACE2) and transmembrane protease serine 2 (TMPRSS2) are two major host factors responsible for the virulence of SARS-CoV-2 and the pathogenesis of SARS-CoV-2. The transmission of SARS-CoV-2 from animals to humans is considered a rare event that necessarily requires strong evolutionary adaptation. To date, apart from ACE2, no other human cell receptors have been found for SARS-CoV-2 entry into human cells [3]. Entry of SARS-CoV-2 into host cells leads to a reaction with the viral spike glycoprotein, which enables the virus to bind to the host cell angiotensin-converting enzyme 2 (ACE2) receptor and accelerates fusion between the host and viral membranes for successful into the host cell [4]. Transmembrane serine protease 2 (TMPRSS2) is a host cell surface protease necessary for viral entry [5]. Recently, ACE2 and TMPRSS2 proteins were found to be expressed in many human organs such as the oral and nasal mucosa, nasopharynx, lung, stomach, small intestine, colon, liver, kidney, and brain, with the most prominent ACE2 protein expression found in alveolar epithelial cells and small intestinal enterocytes. TMPRSS2 expression is higher in the alveolar epithelial cells and prostate epithelium cells than in other human tissues [6,7]. Therefore, the location of ACE2 and TMPRSS2 may provide a possible entry route for SARS-CoV-2. SARS-CoV-2 infection induces a dysregulated immune response, leading to excessive activation of inflammation and the release of inflammatory cytokines in large quantities. An excessive inflammatory response, called cytokine storm, can cause systemic tissue damage, and these factors are directly related to death [8,9].

*Schizophyllum commune* (SC) is a species of fungus in the family Schizophyllaceae and probably the most widespread fungus in existence. SC has been widely consumed by people for a long time, with the effect of maintaining body health and treating neurasthenia. SC is used as a traditional food throughout Southeast Asia and India. Recent studies have shown that SC extracts have potential therapeutic effects including antioxidant, antitumor activity, antivirus, and immunomodulating properties, as well as ACE inhibitory activity [10]. In addition, SC has many bioactive components including schizophyllan, cerebrosides, adenosine, and lectins [11]. Schizophyllan, also known as sizofiran, a neutral exopolysaccharide produced by SC, acts as a biological response modifier and nonspecific stimulator of the immune system. It has been studied to combat many diseases, including acquired immunodeficiency syndrome (AIDS), and to enhance the effectiveness of vaccines and antitumor therapies [12]. Currently, schizophyllan has been commercialized by several pharmaceutical companies as an immunopotentiator and has been used in the clinical treatment of patients receiving antitumor therapy [13]. Additionally, the produces adenosine derivatives such as adenosine, adenosine diphosphate and adenosine 3′,5′-cyclic monophosphate during solid-state culture of SC [11]. Adenosine is a metabolite with immunomodulatory properties that exerts various physiological effects in the cardiovascular system. Adenosine concentrations increase significantly in response to tissue hypoxia or inflammation, and adenosine can significantly reduce the damage caused by cerebral or cardiac ischemia, ischemia/reperfusion injury, tissue inflammation, and sepsis [14]. Adenosine, thus, plays an important role in protecting normal tissues from excessive damage mediated by hypoxia/ischemia and inflammation [13]. In this article, we provide evidence that SC and its constituent adenosine have the potential to be ACE2 and TMPRSS2 inhibitors in vivo and in vitro.

## 2. Results

### 2.1. Cytotoxicity Assessment Was Performed after Exposure to Different Concentrations of SC

**Figure 1:**
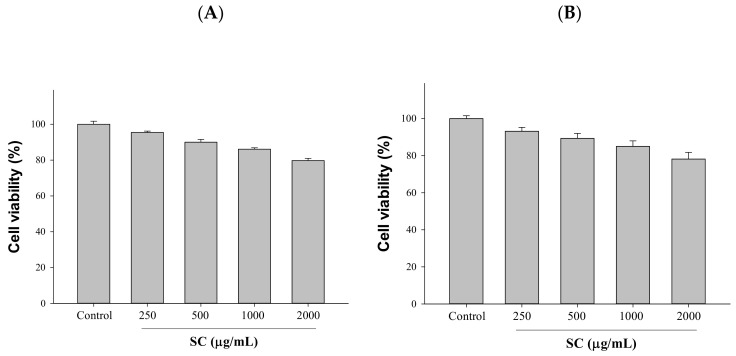
Cytotoxic effects of SC on HepG2 cells and 293T cells: (**A**) HepG2 cells; (**B**) 293T cells. Cells were treated with SC (250, 500, 1000, and 2000 μg/mL) for 24 h, and cytotoxicity was measured using the MTT assay. Data are the means ± SD (*n* = 3).

A high protein expression of ACE2 and TMPRSS2 in HepG2 (hepatocellular carcinoma) and 293T (human embryonic kidney) cells was discovered in a screening model for SARS-CoV-2 entry into host cells [9]. Therefore, the efficacy of SC (250–1000 μg/mL) in ACE2 and TMPRSS2 inhibition in HepG2 cells and 293T cells was investigated. First, we used the MTT method to understand the toxicity of SC toward cells to facilitate subsequent experiments. SC was found to have no toxicity toward cells at concentrations below 1000 μg/mL in MTT experiments; thus, we chose 500 and 1000 μg/mL for subsequent testing in this study (Figure 1).

### 2.2. SC Inhibited ACE2 and TMPRSS2 Expression

**Figure 2:**
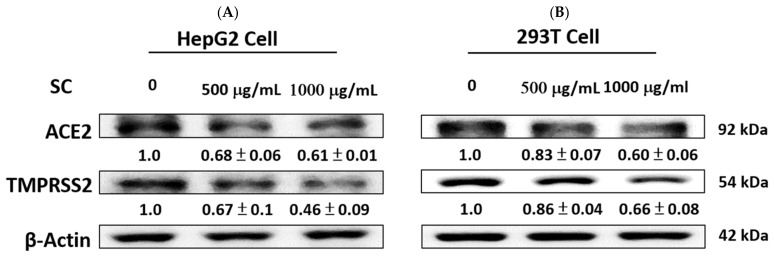
SC inhibited the levels of ACE2 and TMPRSS2 expression in HepG2 cells and 293T cells. HepG2 (**A**) and 293T (**B**) cells were treated with SC (500 and 1000 μg/mL) for 24 h, and the protein expression was measured using Western blot. The densitometric analysis of protein bands is presented as a ratio (SC/control). β-actin was used as the positive control.

As shown in Figure 2, we confirmed that SC affected ACE2 and TMPRSS2 protein expression. SC dose-dependently reduced the levels of ACE2 and TMPRSS2 proteins in HepG2 cells and 293T cells after 24 h.

### 2.3. Cytotoxicity Assessment Was Performed after Exposure to Different Concentrations of Adenosine

**Figure 3:**
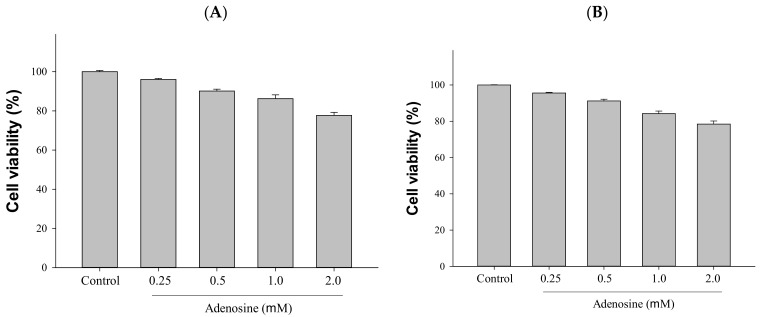
Cytotoxic effects of adenosine on HepG2 cells and 293T cells: (**A**) HepG2 cells; (**B**) 293T cells. Cells were treated with adenosine (0.25–2.0 mM) for 24 h, and cytotoxicity was measured using the MTT assay. Data are the means ± SD (*n* = 3).

To study the function of adenosine in HepG2 and 293T cells, we added different concentrations of adenosine (0.25–2.0 mM) to HepG2 and 293T cells. We performed cell viability assays to estimate the cytotoxicity of adenosine. According to the results, adenosine was found to be nontoxic to cells at concentrations below 1.0 mM in the MTT assay; thus, so we chose 0.5 and 1.0 mM for subsequent assessment of HepG2 and 293T cells (Figure 3).

### 2.4. Adenosine Inhibited ACE2 and TMPRSS2 Expression 

**Figure 4:**
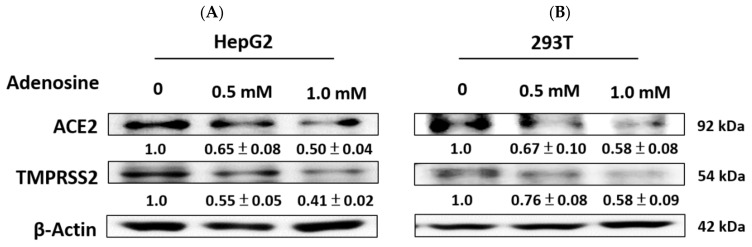
Adenosine inhibited the levels of ACE2 and TMPRSS2 expression in HepG2 cells and 293T cells. HepG2 (**A**) and 293T (**B**) were treated with adenosine (0.5 and 1.0 mM) for 24 h, and ACE2 and TMPRSS2 expression was measured using Western blot. The densitometric analysis of protein bands is presented as a ratio (SC/control). β-actin was used as the positive control.

The efficacy of adenosine (0.5 and 1.0 mM) in inhibiting ACE2 and TMPRSS2 protein expression in cells was investigated. As shown in Figure 4, the data indicate that the treatment of adenosine significantly decreased the expression of ACE2 and TMPRSS2 proteins in HepG2 cells and 293T cells.

### 2.5. Acute Oral Toxicity Study of Schizophyllum Commune 

No lethal effects were found in this test, indicating that the solid-state fermentation of SC was not toxic when administered orally at 5 g/kg. The oral LD_50_ was determined to be >5 g/kg. After 14 days after SC treatment, no mice died, and there were no changes in general appearance or behavioral patterns. Normal weight gain was detected in the SC treatment group and control group. There were no statistically significant differences between the two groups.

### 2.6. The Function of SC in Animal Model

**Figure 5:**
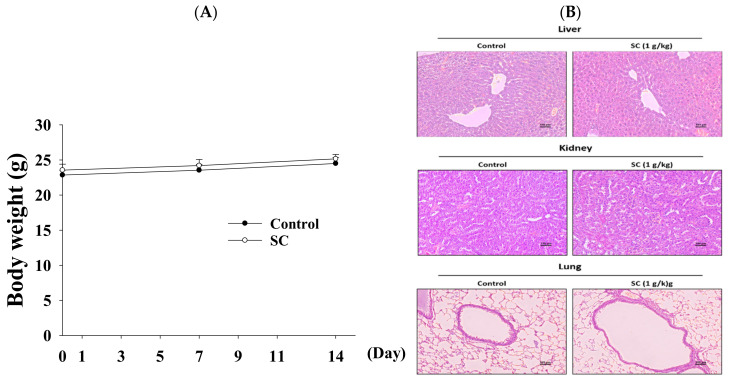
Effects of oral administration of SC in animal models. (**A**) The body weight of mice was calculated after pretreatment with 1.0 g/kg SC. H&E staining images were captured in the liver and kidney tissue (200×) (**B**). The representative histology of mouse liver, kidney, and lung sections after H&E staining is magnified and photographed. Data are the means ± SD (*n* = 6).

In order to study the efficacy of oral SC in vivo, an animal model was established. Mice were pretreated orally with 1.0 g/kg SC for 14 days. The body weight of mice did not change significantly (Figure 5A), and H&E staining of the liver, kidney, and lung showed no toxicity (Figure 5B) after 14 days.

### 2.7. SC Inhibited ACE2 and TMPRSS2 Protein Expression according to Immunohistochemical Images

**Figure 6:**
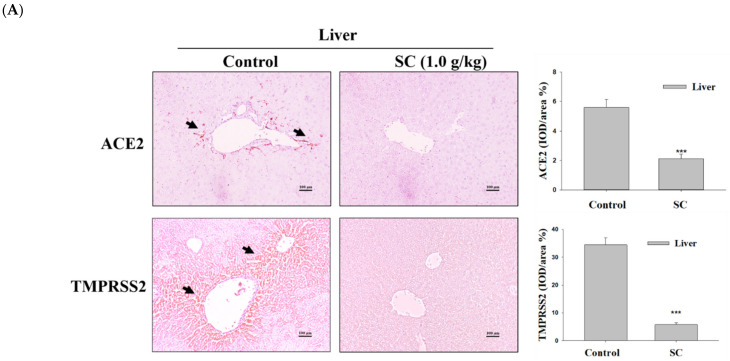

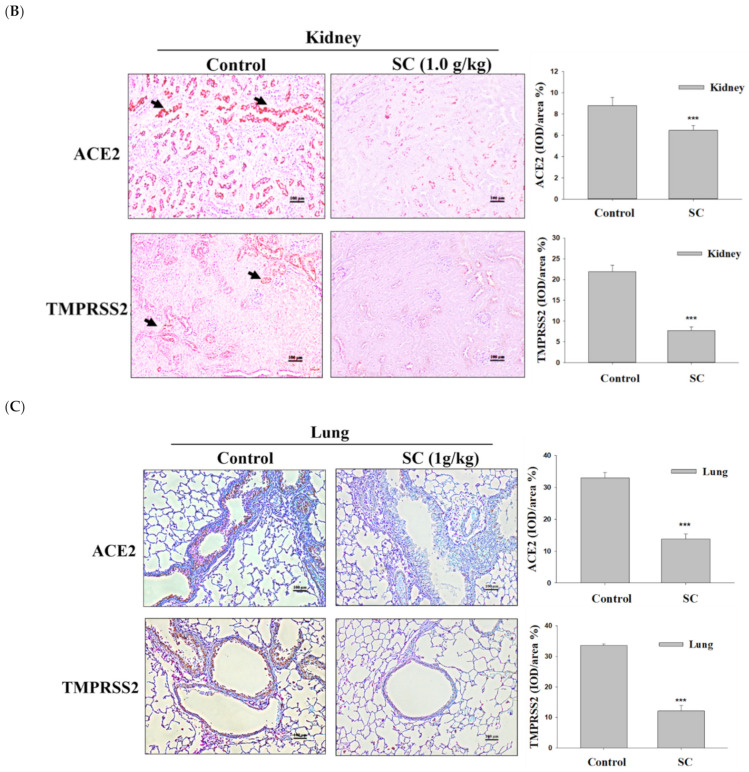
SC inhibits ACE2 and TMPRSS2 expression in an animal model. Oral supplementation of SC 1.0 g/kg SC was administered via oral gavage. Immunohistochemical stained of images captured from the liver (**A**), kidney (**B**), and lung tissue (**C**). The representative histology of mouse liver and kidney sections after H&E staining is magnified and photographed. Data are presented as the IOD/area (%). Data are the mean ± SD (*n* = 6). *** *p* < 0.001 vs. control group. Arrows indicate the ACE2 or TMPRSS2 expression. Scale bar = 100 μm.

Immunohistochemical analysis was used to show whether or not the tissue cells expressed ACE2 and TMPRSS2. The SC (1.0 g/kg) group showed reduced ACE2 and TMPRSS2 expression in the liver, kidney, and lung tissues (Figure 6A,B). Our studies showed that oral supplementation of SC could obstruct the ACE2 and TMPRSS2 protein expression in the liver, kidney, and lung without tissue toxicity.

### 2.8. SC Inhibited ACE2 and TMPRSS2 Protein Expression In Vivo

**Figure 7:**
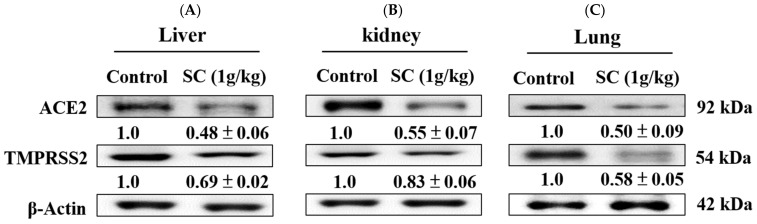
SC inhibited the ACE2 and TMPRSS2 expression in the liver (**A**), kidney (**B**), and lung (**C**) tissues in mice. Protein levels of ACE2 and TMPRSS2 expression in the liver and kidney tissues were assessed using Western blot. The densitometric analysis of protein bands is presented as a ratio (SC/control). Data are the means ± SD (*n* = 3). β-actin was used as a positive control.

We further confirmed whether SC reduced ACE2 and TMPRSS2 expression using Western blotting. Our data show that pretreatment of SC markedly decreased the levels of ACE2 and TMPRSS2 expression in the liver, kidney, and lung tissues (Figure 7A,B).

### 2.9. HPLC Analysis 

**Figure 8:**
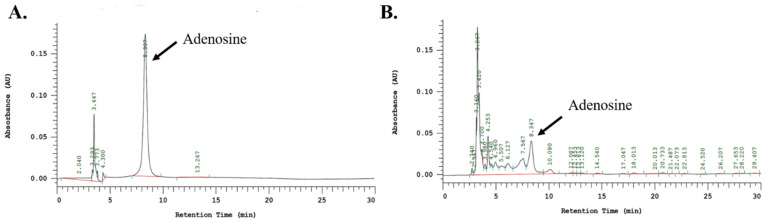
HPLC chromatogram of SC. The highlighted peaks indicate adenosine (8.3 min). HPLC chromatograms of (**A**) adenosine marker substances (**B**) aqueous extract of Schizophyllum commune (SC).

Adenosine was used as a marker of SC. The adenosine content was analyzed using HPLC-PAD (photodiode array detection). As shown in Figure 8A,B, adenosine could be identified by its retention time (8.3 min). The relative content of adenosine in SC was determined to be 14.21 μg/mL.

## 3. Discussion

As the coronavirus disease (SARS-CoV-2) spreads globally, high-risk groups require preventive treatment; thus, adjuvant therapy to reduce viral entry can reduce disease severity and viral spread [15]. Addressing the urgent need for treatment, drug research has been conducted using viral proteins (e.g., remdesivir) or human protein small molecules utilized by viruses, which can be used in conjunction with vaccination to target patients with specific diseases or in delayed vaccinated individuals to prevent possible spread of the virus [16].

Because people infected by SARS-CoV-2 exhibit a wide range of symptoms, efforts have been made to evaluate the expression of ACE2 tissue at the protein level to identify organs that are more vulnerable to infection. Indeed, the expression of ACE2 in the lung is very low, mainly in type II alveolar epithelial cells [17,18]. Because most people have a sore throat after an infection with SARS-CoV-2, this can result in the release of inflammatory factors that can increase ACE2 expression and enhance infection [19]. In addition, the tissues with the highest levels of ACE2 expression were found in the intestinal tract, followed by the kidney, testis, gallbladder, and heart [20]. These results may underlie the gastrointestinal problems and renal dysfunction associated with SARS-CoV-2 infection [21]. Acute kidney injury (AKI) mostly occurs in severe patients with SARS-CoV-2 infection, and ACE2 protein expression is one of the factors influencing AKI [22].

TMPRSS2 triggers the spike protein on the outside of the SARS-CoV-2 particle [23]. Entry of SARS-CoV-2 into respiratory host epithelial cells is mediated by the S1 peptidase domain of TMPRSS2 [24]. It remains to be identified whether inhibition of TMPRSS2 expression mitigates SARS-CoV-2 infection. Camostat, a serine protease inhibitor, is currently used to treat tumors, as well as fight viral infections, liver fibrosis, kidney disease, and pancreatitis. It was discovered to partially block SARS-CoV-2 S-driven entry into cells and is currently active against TMPRSS2 in clinical trials for the treatment of SARS-CoV-2 [25]. Therefore, the identification of inhibitors targeting TMPRSS2 remains important. TMPRSS2 has the highest expression level in the lung, small intestine, kidney, and liver tissue [26]. Studies have found that an important factor in the transmissibility of SARS-CoV-2 is related to the effectiveness of ACE2 and TMPRSS2 [27]. Therefore, the expression of ACE2 and TMPRSS2 plays a crucial role, and they are considered to be the main entry receptors for SARS-CoV-2 infection in humans [28].

SARS-CoV-2 binds to the cell surface receptor ACE2 through the spike protein to help the virus enter host cells, and its S1 domain is triggered by TMPRSS2. This results in uptake of SARS-CoV-2 by host membrane endosomes or fusion of the virus with the host plasma membrane, enabling SARS-CoV-2 entry and host infection [29]. Later, the host renin–angiotensin–aldosterone system (RAAS) is activated, leading to tissue damage characterized by cardiovascular and renal disease [30]. ACE2 is highly expressed in the kidney, especially in the proximal tubule; thus, ACE2 participates in the constriction of blood vessels, as well as the stabilization of cardiac, pulmonary, and renal physiology. However, following infection with SARS-CoV-2, clinical studies have shown a decrease in the expression of ACE2 [31], which may cause an increase in angiotensin II (Ang II) levels and worsen the outcome. When compared with healthy controls, the concentration of Ang II in some patients with SARS-CoV-2 was significantly increased, which was correlated with high virus content and lung damage in patients [32].

The pathological damage of SARS-CoV-2 largely occurs in the lungs and kidneys, but other organs have also been reported, including the liver. The lung is primary target of SARS-CoV-2 infection, which causes respiratory illness. Infection of respiratory epithelial cells with SARS-CoV-2 causes severe coughing, excessive mucus production, shortness of breath, chest tightness, and wheezing. Severe acute respiratory distress syndrome (ARDS) was developed in 17% of SARS-CoV-2 patients in one study, of whom 65% rapidly deteriorated and died due to multiple organ dysfunction syndrome [33]. Lung injury in patients with SARS-CoV-2, including direct viral infection and cellular damage, followed by endothelial barrier disruption, excessive cytokine or chemokine production, and immune cell infiltration, leads to a maladaptive immune response to the virus. SARS-CoV-2 infection can cause kidney damage, especially in people with chronic kidney disease. ACE2 has been identified as the primary receptor for SARS-CoV-2 in the kidney, whereby changes associated with ACE2 expression may be associated with kidney injury during viral infection [34]. Studies have shown kidney damage in more than 30% of hospitalized SARS-CoV-2 patients, and more than 50% of patients in the ICU with renal impairment may require dialysis. The main clinical manifestations of renal injury are increased serum creatinine, proteinuria, and hematuria, along with abnormal renal imaging [35]. Kidney damage caused by SARS-CoV-2 is the result of a variety of causes. SARS-CoV-2 infection of the host initiates the mechanism of kidney injury through the high expression of ACE2 in renal tissue. This renal injury is exacerbated by systemic effects such as impaired host immune tolerance, endothelial cell damage, thrombosis, disturbances in glucose and lipid metabolism, and hypoxia [36]. Many SARS-CoV-2 patients in various studies showed liver enzyme disturbances to varying degrees. Liver damage is mild in most cases, related to the severity of SARS-CoV-2 infection [37]. The cause of liver injury is unknown. Cytokine storm and immune dysregulation may be related, leading to hypoxia, hypotension, and viral infection. In addition, SARS-CoV-2 can cause cytokine storm in lung tissue through immune system dysregulation, resulting in lasting tissue damage [38]. ARDS is also one of the leading causes of death in patients with SARS-CoV-2, mainly triggered by cytokine storm in lung tissues. Currently, clearing SARS-CoV-2 is nearly impossible, and there are no ideal treatments; however, suppressing cellular inflammatory responses may be a viable strategy [39]. SC has shown a very good effect on preventing acute lung injury in the literature.

To fully understand the infectivity of SARS-CoV-2 infection, it is necessary to understand the cell-type-specific expression of host cell surface receptors. The key protein involved in virus entry into the host cell is ACE2, which is expressed in various organs of the human body with inconsistent or conflicting results in some cases. In addition, ACE2 expression is absent in the AT2 lung cancer cell line A549, commonly used as a model for viral replication studies [40]. This article used two cell lines as models for the study. HepG2, a cell line with epithelioid morphology, is a model system suitable for studying highly polarized liver cells. It is used as a model system to study liver metabolism and xenobiotic toxicity, to detect environmental and food cytotoxicity and genotoxicity, as well as liver cancer, and in drug targeting studies. The human embryonic kidney 293T cell line is derived from human embryonic kidney (HEK) 293 cells containing the simian virus 40 (SV40) large T antigen. The 293T cells are mainly used to study cell tissue culture, DNA replication, gene expression and protein production, and antiviral drugs [41]. Therefore, this study utilized these two cell lines for antiviral experiments. The present study showed that SC and adenosine obviously reduced the ACE2 and TMPRSS2 expression in cells. In an animal model, there were no significant differences in body weight or H&E staining of oral SC compared to controls. In addition, in the control group, ACE2 and TMPRSS2 were highly expressed in the liver and kidney tissues, whereas, in the SC group, expression was significantly reduced in both liver and kidney, as shown by IHC staining. These findings provide evidence that SC can be used as a possible food nutritional supplement to suppress SARS-CoV-2 infection.

SC is a filamentous fungus that can be found all over the world. This mushroom is known for its various biological and medicinal activities and has been widely used in Japan, China, and Taiwan. SC has medicinal properties, including antioxidant, cardioprotective, antitumor, anti-inflammatory, and immunomodulatory properties [42]. Fungi use fermentation processes to produce secondary metabolites. There are currently two technologies for the production of secondary metabolites, liquid fermentation and solid-state fermentation (SSF). Submerged fermentation involves the production of microorganisms in a liquid nutrient medium on enzymes. SSF is a process that takes place on insoluble matter that acts as a support and source of nutrients. This process consumes less water, and the material is fermented. In contrast to other microorganisms, fungi usually grow on solid substrates. This is why solid-state fermentation is best used when fungi are present [43]. The material utilized in this paper was derived from the production of SC by SSF. SSF represents an artificial simulation of the growth of fungal cells that can be used to produce enzymes, ethanol, organic acids, and vitamin products, and it is one of the most common methods in the food industry. The SSF technology threshold has strong development potential due to its low energy requirements and low infrastructure investment [44]. In previous pharmacological studies, SC was also demonstrated to suppress cytokine production (interferon-γ, tumor necrosis factor-α, interleukin (IL)-1β, IL-6, IL-8, and IL-10) in dengue-infected human monocytes infected with dengue virus-2 (DENV-2) New guinea C strain [43], regulate immune responses in chronic hepatitis B patients [45], and scavenge free radicals [46]. 

Schizophyllan has numerous biological activities that are widely used in medicine, including antitumor, antibacterial, antiparasitic, hepatoprotective, and anti-inflammatory properties [47]. The composition and biological activity of schizophyllan is similar to that of lentinan, and the mechanisms of its immunomodulatory and antitumor effects appear to be very similar [48]. Schizophyllan is a T-cell-oriented immune enhancer that increases the production of helper T cells; increased production of macrophages leads to a non-immune increase in host defense mechanisms, which in turn increases the cell proliferation of macrophages, neutrophils, and lymphocytes, as well as activates the complement system [49].

Adenosine is a primary nucleoside for building RNA and DNA, and it has different derivatives, including adenosine monophosphate (AMP), adenosine diphosphate (ADP), and adenosine triphosphate (ATP). These derivatives act as signal transductions for the modulation of different physiological processes. Adenosine acts as a cytoprotective signal against tissue injury. Adenosine has immunosuppressive and anti-inflammatory effects and is upregulated during ischemia and tissue hypoxia [14]. In addition, adenosine is a non-human cell endogenous adenosine, which directly enters the myocardium through phosphorylation to generate cyclic adenosine monophosphate (cAMP) and participates in myocardial energy metabolism. At the same time, adenosine is also involved in the expansion of coronary arteries, increasing blood flow, and has obvious physiological effects in many tissues [50]. Adenosine has also been shown to block polymorphic nuclear (PMN) infiltration, activation, and superoxide production, thereby attenuating reperfusion injury [51]. Considering these pharmacological activities, adenosine can be used in the treatment of acute lung injury (ALI) and ARDS [51]. Adenosine was shown to diminish inflammation and modulate endothelial integrity in models of ALI and ARDS [52]. In a clinical study investigating treatment of SARS-CoV-2 patients with inhaled adenosine, it was found that the use of inhaled adenosine in SARS-CoV-2 patients could reduce the average length of hospital stay by 6 days. Significant improvements in PaO_2_/FiO_2_ and reductions in inflammatory parameters were observed in treated patients. This treatment appears to be safe and modulates the immune system to respond effectively to viral infection progression, reducing hospital stay and inflammatory parameters [1]. Therefore, the adenosine holds promise as a food nutritional for SARS-CoV-2.

## 4. Materials and Methods

### 4.1. Materials

SC was obtained from Tsairder Biotechnology Co. Ltd., Taichung, Taiwan. The dried SC (100 g) was ground to pass through 80 mesh and extracted using distilled water at 65 °C, for up to 1 h. The extract was filtered using a rotary evaporator at 40 ± 5 °C under vacuum, which yielded stable freeze-dried powders that were stored at −20 °C until use. 

### 4.2. Cell Culture and Treatment

Human HepG2 and 293T cells were provided by the Bioresource Collection and Research Center (Hsinchu, Taiwan). Cells were grown in Dulbecco’s modified Eagle medium (DMEM) supplemented with 10% fetal bovine serum at 37 °C in a humidified atmosphere at 5% CO_2_. A total of 2.5 × 10^4^ cells were seeded into six-well plates. Before the start of each experiment, cells were cultured in DMEM for 24 h and subjected to various treatments.

### 4.3. Cell Viability

Cell lines were plated in 96-well plates at 2.5 × 10^4^ cells/well and treated with various concentrations of SC (0–1000 μg/mL) and adenosine (0–2 mM) for 24 h. Cell viability was examined using an MTT assay kit (Med-ChemExpress, HY-15924) and then incubated for at least 3 h. The absorbance was measured on an ELISA plate reader (Molecular Devices, San Jose, CA, USA) at a wavelength of 570 nm.

### 4.4. Western Blot Analysis

Total protein was extracted from cells or tissue by homogenization in RIPA buffer (GENESTAR, Kaohsiung, Taiwan). The homogenates were then centrifuged at 10,000× *g* for 10 min at 4 °C. The supernatant was quantified using a Bio-Rad protein assay kit (BioRad, Hercules, CA, USA). Samples with equal amounts of protein were separated within a gel (100 V for 90 min) and transferred to a membrane at 200 mA for 2 h. The blocked membrane was then incubated with primary antibodies (ACE2: GTX101395, dilution 1500×; TMPRSS2: GTX100743, dilution 1500×; Genetex, San Antonio, TX, USA) and secondary antibodies (goat anti-rabbit IgG antibody (HRP): ARG65351, dilution 5000×; Arigo, Hsinchu, Taiwan). Detection was performed using an ECL substrate (201765; Merck, Branchburg, NJ, USA). Protein expression was calculated using Kodak Molecular Imaging Software (East-man Kodak Company, Rochester, NY, USA).

### 4.5. Acute Oral Toxicity

Male and female ICR mice (6–8 weeks old, weight 25–30 g) were acquired from BioLASCO Taiwan Co., Ltd. (Taipei, Taiwan). Mice were randomly assigned to two groups (*n* = 5): Group I, normal control (received only distilled water); Group II, 5.0 g/kg SC. The behavior of mice and clinical signs of toxicity were recorded during the experimental period. Animals were observed every hour on the first day, and then observed every day for 14 days. Animal care and experiments complied with the Animal Management Committee of China Medical University (IACUC approval number: CMUIACUC-2020-358).

### 4.6. Mouse Model

Female C_57_BL/_6_ mice (6–8 weeks old, weight 18–20 g) were obtained from BioLASCO Taiwan Co. Mice were randomly divided into two groups (*n* = 6): Group I, normal control (received only distilled water); Group II, 1.0 g/kg SC by oral gavage for 14 days. The body weight of mice was calculated on days 0, 7, and 14. After 14 days, tissues were collected from the mice, and they were sacrificed. Animal care and experiments complied with the Animal Management Committee of China Medical University (IACUC approval number: ǺCMUIACUC-2022-127). 

### 4.7. Histopathological Analysis

Tissues were preserved in formalin, embedded in a paraffin substrate, sectioned, and stained with hematoxylin and eosin (H&E) to evaluate morphology and damage. Sections were observed and photographed under a microscope (Nikon, ECLIPSE, TS100, Tokyo, Japan).

### 4.8. Immunohistochemistry (IHC)

Tissues were preserved in formalin, embedded in a paraffin substrate, sectioned, and incubated with antibodies specific for ACE2 (bs-1004R, Bioss Inc. MA, USA, 1:50) or TMPRSS2 (ab214462, Abcam; MA, USA, 1:200). Sections were observed and photographed under a microscope (Nikon, ECLIPSE, TS100, Tokyo, Japan). Stained areas were quantified using Image J software.

### 4.9. Determination of Adenosine by HPLC

The composition of SC was analyzed using HPLC (Hitachi Ltd., Tokyo, Japan). The eluted fractions were determined as a function of the retention time compared to the reference standard used. The identification of the components was also confirmed using a photodiode array detector by comparison to standard UV spectra at a wavelength of 254 nm. Compound separations were achieved on a 250 × 4.6 mm i.d., 5 µm reversed-phase TSKgel Tosoh ODS-80Tm column (Tosoh, Yamaguchi, Japan) with acetonitrile (A) and 0.4% acetic acid (B) as the mobile phases, according to the following linear gradient: 0–20 min, 2–10% A, 98–90% B; 20–30 min, 10–25% A, 90–75% B; 30–35 min, 25–2% A, 75–98% B; 35–40 min, 2–2% A, 98–98% B. The flow rate was 1 mL/min.

### 4.10. Statistical Analyses

All data are shown as the mean ± standard deviation (SD). Data were analyzed using SPSS software 21.0 (SPSS, Inc., Chicago, IL, USA). Two groups were analyzed using the unpaired two-tailed Student’s *t*-test; more than two groups were analyzed using the one-way analysis of variance (ANOVA) and Scheffé test. A *p*-value < 0.05 was considered statistically significant.

## 5. Conclusions

In this article, we showed that SC and adenosine could diminish the ACE2 and TMPRSS2 expression in a cellular model and mouse organs. Thus, SC and adenosine potentially have the ability to suppress the infection rates or propagation of SARS-CoV-2.

## Data Availability

Data are contained within the article.

## Sample Availability

Samples of the crude extracts prepared from solid state fermentation of Schizophyllum commune are available from the authors.

## References

[1] Datta P.K., Liu F., Fischer T., Rappaport J., Qin X. (2020). SARS-CoV-2 pandemic and research gaps: Understanding SARS-CoV-2 interaction with the ACE2 receptor and implications for therapy. Theranostics.

[2] Zhang X., Li S., Niu S. (2020). ACE2 and COVID-19 and the resulting ARDS. Postgrad. Med. J..

[3] Li L.Q., Huang T., Wang Y.Q., Wang Z.P., Liang Y., Huang T.B., Zhang H.Y., Sun W., Wang Y. (2020). COVID-19 patients’ clinical characteristics, discharge rate, and fatality rate of meta-analysis. J. Med. Virol..

[4] Singh M.K., Mobeen A., Chandra A., Joshi S., Ramachandran S. (2021). A meta-analysis of comorbidities in COVID-19: Which diseases increase the susceptibility of SARS-CoV-2 infection?. Comput. Biol. Med..

[5] Zamorano Cuervo N., Grandvaux N. (2020). ACE2: Evidence of role as entry receptor for SARS-CoV-2 and implications in comorbidities. Elife.

[6] Hoffmann M., Kleine-Weber H., Schroeder S., Krüger N., Herrler T., Erichsen S., Schiergens T., Herrler G., Wu N., Nitsche A. (2020). SARS-CoV-2 cell entry depends on ACE2 and TMPRSS2 and is blocked by a clinically proven protease inhibitor. Cell.

[7] Dong M., Zhang J., Ma X., Tan J., Chen L., Liu S., Xin Y., Zhuang L. (2020). ACE2, TMPRSS2 distribution and extrapulmonary organ injury in patients with COVID-19. Biomed. Pharmacother..

[8] Wu C.-Y., Lin Y.-S., Yang Y.-H., Shu L.-H., Cheng Y.-C., Te Liu H. (2020). GB-2 inhibits ACE2 and TMPRSS2 expression: In vivo and in vitro studies. Biomed. Pharmacother..

[9] Qi J., Zhou Y., Hua J., Zhang L., Bian J., Liu B., Zhao Z., Jin S. (2021). The scRNA-seq expression profiling of the receptor ACE2 and the cellular protease TMPRSS2 reveals human organs susceptible to SARS-CoV-2 infection. Int. J. Environ. Res. Public Health.

[10] Mišković J., Karaman M., Rašeta M., Krsmanović N., Berežni S., Jakovljević D., Piattoni F., Zambonelli A., Gargano M.L., Venturella G. (2021). Comparison of Two Schizophyllum commune Strains in Production of Acetylcholinesterase Inhibitors and Antioxidants from Submerged Cultivation. J. Fungi.

[11] Patel S. (2019). Immunomodulatory aspects of medicinal mushrooms. Medicinal Mushrooms.

[12] Leathers T.-D., Nunnally M.-S., Price N.-P. (2006). Co-production of schizophyllan and arabinoxylan from corn fiber. Biotechnol Lett..

[13] Wong J.-H., Ng T.-B., Chan H.-H.-L., Liu Q., Man G.-C.-W., Zhang C.-Z., Guan S., Ng C.-C.-W., Fang E.-F., Wang H. (2020). Mushroom extracts and compounds with suppressive action on breast cancer: Evidence from studies using cultured cancer cells, tumor-bearing animals, and clinical trials. Appl. Microbiol. Biotechnol..

[14] Raskovalova T., Lokshin A., Huang X., Su Y., Mandic M., Zarour H.-M., Jackson E.-K., Gorelik E. (2007). Inhibition of cytokine production and cytotoxic activity of human antimelanoma specific CD^8+^ and CD^4+^ T lymphocytes by adenosine-protein kinase A type I signaling. Cancer Res..

[15] Naidu A.S., Shahidi F., Wang C.-K., Sato K., Wirakartakusumah A., Aworhf O.C., Halliwell B., Clemensh R.A. (2022). SARS-CoV-2-induced Host Metabolic Reprogram (HMR): Nutritional Interventions for Global Management of COVID-19 and Post-Acute Sequelae of COVID-19 (PASC). J. Food Bioact..

[16] Dhama K., Sharun K., Tiwari R., Dadar M., Malik Y.S., Singh K.P., Chaicumpa W. (2020). COVID-19, an emerging coronavirus infection: Advances and prospects in designing and developing vaccines, immunotherapeutics, and therapeutics. Hum. Vaccines Immunother..

[17] Ziegler C.G., Allon S.J., Nyquist S.K., Mbano I.M., Miao V.N., Tzouanas C.N., Cao Y., Yousif A.S., Bals J., Hauser B.M. (2020). SARS-CoV-2 receptor ACE2 is an interferon-stimulated gene in human airway epithelial cells and is detected in specific cell subsets across tissues. Cell.

[18] Stewart C.A., Gay C.M., Ramkumar K., Cargill K.R., Cardnell R.J., Nilsson M.B., Heeke S., Park E.M., Kundu S.T., Diao L. (2020). SARS-CoV-2 infection induces EMT-like molecular changes, including ZEB1-mediated repression of the viral receptor ACE2, in lung cancer models. BioRxiv.

[19] Nugraha R.V., Ridwansyah H., Ghozali M., Khairani A.F., Atik N. (2020). Traditional herbal medicine candidates as complementary treatments for COVID-19: A review of their mechanisms, pros and cons. Evid.-Based Complement. Altern. Med..

[20] Zhou L., Niu Z., Jiang X., Zhang Z., Zheng Y., Wang Z., Zhu Y., Gao L., Huang H., Wang X. (2020). Systemic analysis of tissue cells potentially vulnerable to SARS-CoV-2 infection by the protein-proofed single-cell RNA profiling of ACE2, TMPRSS2 and Furin proteases. BioRxiv.

[21] Steiger S., Rossaint J., Zarbock A., Anders H.-J. (2022). Secondary immunodeficiency related to kidney disease (SIDKD)—Definition, unmet need, and mechanisms. J. Am. Soc. Nephrol..

[22] Gabarre P., Dumas G., Dupont T., Darmon M., Azoulay E., Zafrani L. (2020). Acute kidney injury in critically ill patients with COVID-19. Intensive Care Med..

[23] Rahman N., Basharat Z., Yousuf M., Castaldo G., Rastrelli L., Khan H. (2020). Virtual screening of natural products against type II transmembrane serine protease (TMPRSS2), the priming agent of coronavirus 2 (SARS-CoV-2). Molecules.

[24] Sun Y.J., Velez G., Parsons D.E., Li K., Ortiz M.E., Sharma S., McCray P.B., Bassuk A.G., Mahajan V.B. (2021). Structure-based phylogeny identifies avoralstat as a TMPRSS2 inhibitor that prevents SARS-CoV-2 infection in mice. J. Clin. Investig..

[25] Sharma V., Sharma A., Bharate S.B. (2021). Natural products in mitigation of SARS CoV Infections. Curr. Med. Chem..

[26] Chen J., Fan J., Chen Z., Zhang M., Peng H., Liu J., Ding L., Liu M., Zhao C., Zhao P. (2021). Nonmuscle myosin heavy chain IIA facilitates SARS-CoV-2 infection in human pulmonary cells. Proc. Natl. Acad. Sci. USA.

[27] Ozono S., Zhang Y., Ode H., Sano K., Tan T.S., Imai K., Miyoshi K., Kishigami S., Ueno T., Iwatani Y. (2021). SARS-CoV-2 D614G spike mutation increases entry efficiency with enhanced ACE2-binding affinity. Nat. Commun..

[28] Bourgonje A.R., Abdulle A.E., Timens W., Hillebrands J.L., Navis G.J., Gordijn S.J., Bolling M.C., Dijkstra G., Voors A.A., Osterhaus A.D. (2020). Angiotensin-converting enzyme 2 (ACE2), SARS-CoV-2 and the pathophysiology of coronavirus disease 2019 (COVID-19). J. Pathol..

[29] Yan R., Zhang Y., Li Y., Xia L., Guo Y., Zhou Q. (2020). Structural basis for the recognition of SARS-CoV-2 by full-length human ACE2. Science.

[30] Lumbers E.R., Head R., Smith G.R., Delforce S.J., Jarrott B., Martin J.H., Pringle K.G. (2022). The interacting physiology of COVID-19 and the renin-angiotensin-aldosterone system: Key agents for treatment. Pharmacol. Res. Perspect..

[31] Shukla A.K., Banerjee M. (2021). Angiotensin-converting-enzyme 2 and renin-angiotensin system inhibitors in COVID-19: An update. High Blood Press Cardiovasc Prev..

[32] Cure M.C., Cure E. (2022). Prolonged NHE activation may be both cause and outcome of cytokine release syndrome in COVID-19. Curr. Pharm. Des..

[33] Wang M., Xiong H., Chen H., Li Q., Ruan X.Z. (2021). Renal injury by SARS-CoV-2 infection: A systematic review. Kidney Diseases.

[34] Ghoda A., Ghoda M. (2020). Liver injury in COVID-19 infection: A systematic review. Cureus.

[35] Saeedi-Boroujeni A., Mahmoudian-Sani M.-R. (2020). COVID-19 Pandemic along with Pandemic of Lifestyle-Associated Diseases Victimizes Patients in an Inflammation Context!. Dubai Med. J..

[36] Azkur A.K., Akdis M., Azkur D., Sokolowska M., van de Veen W., Brüggen M.C., O’Mahony L., Gao Y., Nadeau K., Akdis C.A. (2020). Immune response to SARS-CoV-2 and mechanisms of immunopathological changes in COVID-19. Allergy.

[37] Brendler T., Al-Harrasi A., Bauer R., Gafner S., Hardy M.L., Heinrich M., Hosseinzadeh H., Izzo A.A., Michaelis M., Nassiri-Asl M. (2021). Botanical drugs and supplements affecting the immune response in the time of COVID-19: Implications for research and clinical practice. Phytother. Res..

[38] Dianat N., Steichen C., Vallier L., Weber A. (2013). Dubart-Kupperschmitt, A., Human pluripotent stem cells for modelling human liver diseases and cell therapy. Curr. Gene Ther..

[39] Vora S.-M., Lieberman J., Wu H. (2021). Inflammasome activation at the crux of severe COVID-19. Nat. Rev. Immunol..

[40] Hikmet F., Méar L., Edvinsson Å., Micke P., Uhlén M., Lindskog C. (2020). The protein expression profile of ACE2 in human tissues. Mol. Syst. Biol..

[41] Españo E., Kim J., Lee K., Kim J.-K. (2021). Phytochemicals for the treatment of COVID-19. J. Microbiol..

[42] Thongsiri C., Nagai-Yoshioka Y., Yamasaki R., Adachi Y., Usui M., Nakashima K., Nishihara T., Ariyoshi W. (2021). Schizophyllum commune β-glucan: Effect on interleukin-10 expression induced by lipopolysaccharide from periodontopathic bacteria. Carbohydr. Polym..

[43] Ellan K., Thayan R., Phan C., Sabaratnam V. (2019). Anti-inflammatory effect of mushrooms in dengue-infected human monocytes. Trop. Biomed..

[44] Hobbs C. (2005). The chemistry, nutritional value, immunopharmacology, and safety of the traditional food of medicinal split-gill fugus *Schizophyllum commune* Fr.: Fr.(Schizophyllaceae). A literature review. Int. J. Med. Mushrooms.

[45] Kakumu S., Ishikawa T., Wakita T., Yoshioka K., Ito Y., Shinagawa T. (1991). Effect of sizofiran, a polysaccharide, on interferon gamma, antibody production and lymphocyte proliferation specific for hepatitis B virus antigen in patients with chronic hepatitis B. Int. J. Immunopharmacol..

[46] Mirfat A.-H.-S., Noorlidah A., Vikineswary S. (2010). Scavenging activity of Schizophyllum commune extracts and its  correlation to total phenolic content. J. Trop. Agric. Food Sci..

[47] Geiger J.-D., Khan N., Murugan M., Boison D. (2020). Possible role of adenosine in COVID-19 pathogenesis and therapeutic opportunities. Front. Pharmacol..

[48] Ren L., Perera C., Hemar Y. (2012). Antitumor activity of mushroom polysaccharides: A review. Food Function.

[49] Mochizuki S., Miyamoto N., Sakurai K. (2022). Oligonucleotide delivery to antigen presenting cells by using schizophyllan. Drug Metab. Pharmacokinet..

[50] Falcone C., Caracciolo M., Correale P., Macheda S., Vadalà E.G., La Scala S., Tescione M., Danieli R., Ferrarelli A., Tarsitano M.G. (2020). Can adenosine fight COVID-19 acute respiratory distress syndrome?. J. Clin. Med..

[51] Lucas R., Verin A.D., Black S.M., Catravas J.D. (2009). Regulators of endothelial and epithelial barrier integrity and function in acute lung injury. Biochem. Pharmacol..

[52] Li X., Berg N.-K., Mills T., Zhang K., Eltzschig H.-K., Yuan X. (2021). Adenosine at the interphase of hypoxia and inflammation in Lung Injury. Front. Immunol..

